# Machine Learning-Based Fast Banknote Serial Number Recognition Using Knowledge Distillation and Bayesian Optimization

**DOI:** 10.3390/s19194218

**Published:** 2019-09-28

**Authors:** Eunjeong Choi, Somi Chae, Jeongtae Kim

**Affiliations:** Department of Electronics and Electrical Engineering, Ewha Womans University, Seoul 03760, Korea; ejeong_choi@naver.com (E.C.); somi6002@gmail.com (S.C.)

**Keywords:** banknote serial number recognition, deep learning, knowledge distillation

## Abstract

We investigated a machine-learning-based fast banknote serial number recognition method. Unlike existing methods, the proposed method not only recognizes multi-digit serial numbers simultaneously but also detects the region of interest for the serial number automatically from the input image. Furthermore, the proposed method uses knowledge distillation to compress a cumbersome deep-learning model into a simple model to achieve faster computation. To automatically decide hyperparameters for knowledge distillation, we applied the Bayesian optimization method. In experiments using Japanese Yen, Korean Won, and Euro banknotes, the proposed method showed significant improvement in computation time while maintaining a performance comparable to a sequential region of interest (ROI) detection and classification method.

## 1. Introduction

Correct recognition of banknote serial numbers is important because it can be used to trace the circulation routes of individual banknotes to provide essential information about economic activities [1,2]. For this reason, many investigations of serial number recognition have been conducted [2,3,4,5,6,7]. The serial number recognition problem essentially belongs to the printed character recognition problem which has been studied in other fields such as license plate recognition [8] and address recognition [9]. Unlike these other fields, serial number recognition for banknotes requires extremely high accuracy since even a small error can result in a huge financial loss [2]. In addition, real-time recognition must meet the required transaction times for teller machines. Consequently, banknote serial number recognition requires the design of a fast serial number recognition system with very high accuracy, a challenging task.

Typical serial number recognition systems execute a pre-processing step and a character recognition step [1]. The first step includes detection of the region of interest (ROI) that contains the serial number in the acquired banknote image as well as enhancement of the acquired image [1]. ROI detection is challenging for various reasons including uneven illumination, smears, and patterns present in the background [2]. To resolve these problems, several methods have been investigated using image processing techniques [10]. After extracting a region for the serial number, segmentation of the serial number is implemented to classify each digit individually [2]. To the best of our knowledge, all reported methods execute single digit classification after segmentation of each digit [2,4,11]. However, it has been reported that individual character classification usually requires longer computation times than simultaneous classification of all digits [12].

The second step is character recognition in which the segmented characters are identified [2]. Many methods for character recognition have been investigated [2,13,14,15,16,17,18]. One may divide this step into the two sub-steps of feature extraction and classification as was done in a previous investigation [2]. The feature extraction step attempts to extract the most useful features of a single digit’s image in order to improve character classification. These may include a histogram of oriented gradients (HOG), intensity values, or Gabor features [2,6,13,14]. The classification step classifies the image of each digit using the features extracted in the previous step [2]. Several different classification methods have been investigated including support vector machines (SVM), convolutional neural networks (CNN), hybrid CNN-SVM, and the modified quadratic discriminant function (MQDF) [2,4,6,17,18]. Although many methods have been proposed and studied, we believe that existing methods need to be improved in terms of performance and learning capability. It is desirable for a serial number recognition method to be able to learn how to correctly recognize a previously unrecognizable banknote. We propose that a machine learning-based method is one of the best candidates to accomplish that since it can be retrained whenever an unrecognized number occurs; it does not require the redesign of complicated character segmentation and classification methods. Recently, many deep-learning-based methods have been successfully applied to banknote recognition [19,20,21].

To meet the requirements discussed above, we propose a fast high-performance banknote serial number recognition system based on deep-learning technology. In this paper, we assume that the input image of the system is not rotated since it can be automatically aligned in an image acquisition system. If the input image is significantly distorted, it is difficult to detect ROI and to classify it correctly. In this event, we can consider rotation-invariant methods [22,23]. Unlike conventional methods, the proposed method processes every recognition step using machine learning-based methods. In other words, we not only classify characters in an extracted serial number region but also determine the serial number region using a machine learning-based method. Moreover, we recognize the entire serial number at once to achieve a faster computation time. Please note that the evaluation of one shallow network can be much faster than sequential evaluation as reported in a previous investigation [12]. In addition, we attempt to further reduce computation time through concurrent detection of ROI and classification of characters, unlike existing methods that sequentially process ROI detection and character classification. Furthermore, we attempt to make the recognition even faster by compressing the joint model using knowledge distillation [12,24], one of the most promising of the model compression methods that attempt to reduce complicated machine learning systems into simple systems [25]. Because there are many hyperparameters in knowledge distillation-based model compression, their determination is cumbersome. To overcome this problem, we apply a Bayesian optimization method to automatically determine hyperparameters. The Bayesian optimization method is useful in optimizing parameters for a system that is non-differentiable and computationally heavy to evaluate [26,27,28]. In addition, the Bayesian optimization method showed better performance than genetic algorithms in [29] and required less computation than the genetic algorithms as the complexity of the problem increased [29]. Previous studies have applied Bayesian optimization in various areas to determine hyperparameters [30,31,32]. We verify the performance of the proposed method through experiments using Japanese Yen, Korean Won, and Euro banknotes.

The rest of this paper is organized as follows. In Section 2, we review related works and in Section 3, we explain three important aspects of the proposed method in detail: joint regression and classification, knowledge distillation, and Bayesian optimization. In Section 4, we demonstrate the usefulness of the proposed method using four data sets taken from Japanese Yen, Korean Won, and Euro banknotes. We include discussion and conclusions in the following sections.

## 2. Related Work

**Figure 1:**
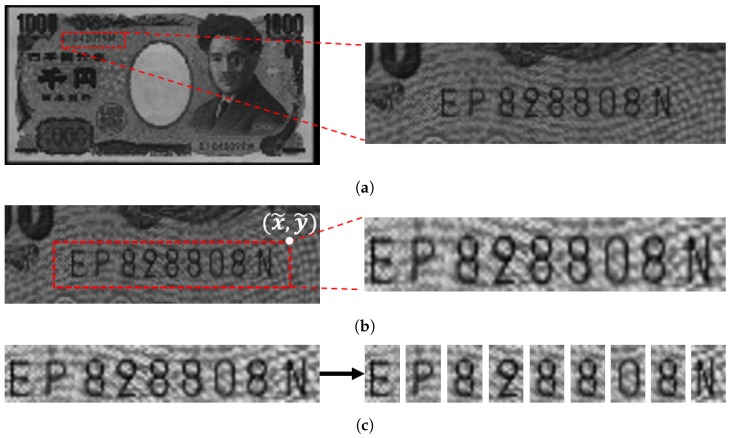
Images of Japanese Yen and their cropped image. (**a**) Region detection (**b**) ROI detection (**c**) Character classification.

**Figure 2:**
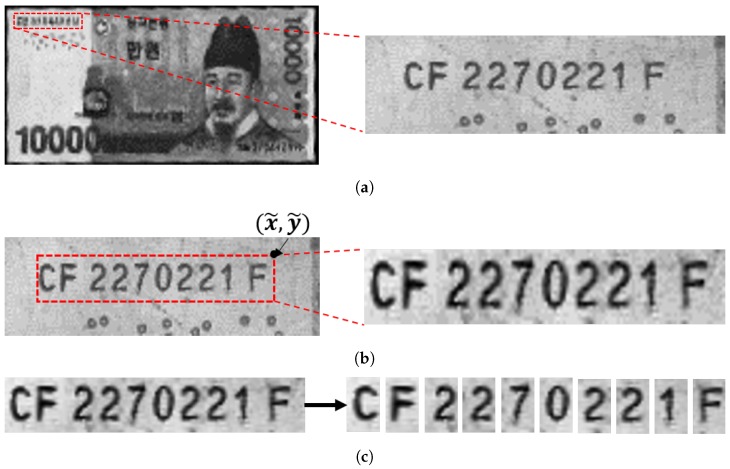
Images of Korean Won and their cropped image. (**a**) Region detection (**b**) ROI detection (**c**) Character classification.

**Figure 3:**
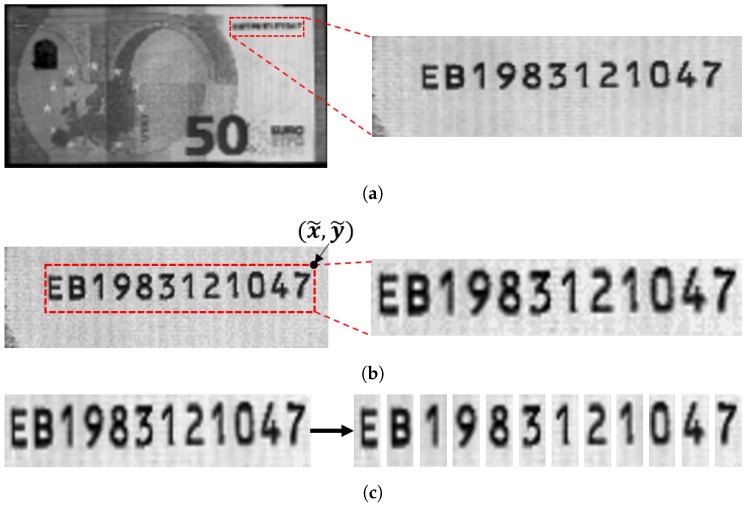
Images of Euro and their cropped image. (**a**) Region detection (**b**) ROI detection (**c**) Character classification.

For banknote recognition, images of an incoming banknote are acquired using various sensors including visible light and infrared sensors [1]. Assuming that the variation of angles and locations of the images is small, a certain section of the incoming image is cropped and sent for serial number recognition. From the cropped image, a bounding box for the serial number is usually determined. Using the bounding box, most conventional methods segment each digit and decode each digit sequentially [9]. In this paper, we denote these steps as region detection, ROI detection, and character classification, respectively. Figure 1, Figure 2 and Figure 3 show images of Japanese Yen, Korean Won, and Euro banknotes and their cropped images, respectively.

As explained above, a typical serial number recognition system classifies each digit sequentially. To recognize each digit correctly, traditional methods extract hand-crafted features such as HOG, intensity, or Gabor features [2,6,13,14]. The extracted features are applied to classifiers such as MQDF and SVM [2,6,14]. However, hand-crafted feature-based approaches are time-consuming, complicated and require good knowledge of input data. Recently, to solve these problems, CNN-based character recognition methods that can automatically extract features from input images have been investigated [15,16]. Some methods introduce a hybrid CNN-SVM classifier for character recognition, where CNN and SVM are used as a feature extractor and a classifier, respectively [2,17,18].

**Figure 4:**
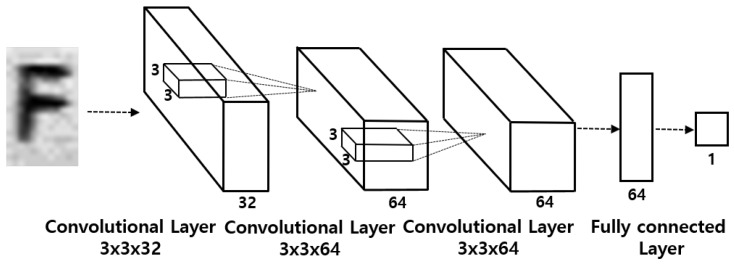
A CNN-based single digit classification system.

We implemented a CNN-based single digit classification system as shown in Figure 4. We call this method the single digit CNN (*sd_cnn*) method in this paper. We also implemented a hybrid CNN-SVM-based single digit classification system and we call this the single digit CNN-SVM (*sd_cnn_svm*) method in this paper. To implement the hybrid CNN-SVM model, we first trained the CNN as shown in Figure 4, then trained the SVM model using the features extracted from the trained CNN model. Although the *sd_cnn* and *sd_cnn_svm* methods perform satisfactorily [2,17], we think that these methods need to be improved because they usually require longer computation time than the simultaneous classification of all digits [12]. To shorten the computation, we propose a machine learning method that recognizes the entire serial number simultaneously with high accuracy.

## 3. Methodology

### 3.1. Joint Regression and Classification Machine Learning System

**Figure 5:**
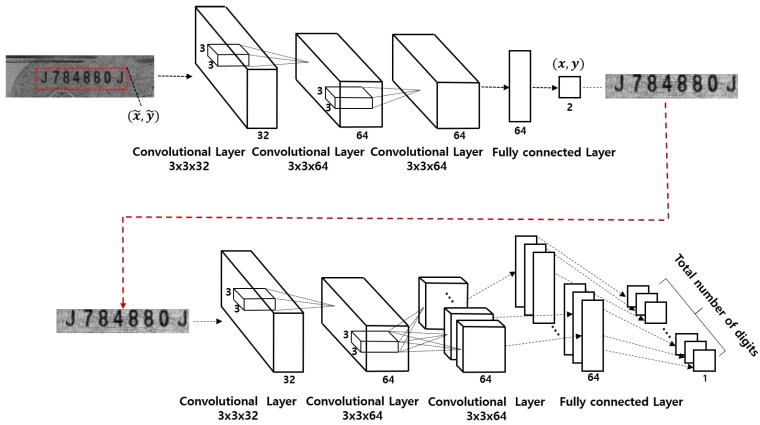
The sequential ROI detection and classification system.

To recognize the serial number digits simultaneously, we first designed a sequential ROI detection and classification system as shown in Figure 5. In the figure, the first CNN (the ROI detection CNN) detects the ROI for the serial number. Using the detected ROI, the second CNN (the classification CNN) classifies all characters in the ROI simultaneously. Because the size of the ROI is pre-determined depending on the kind of banknote and the input image is assumed not to be tilted, it is only necessary to detect a single point in the ROI. In this research, we used the upper right corner of the bounding box that contains the entire serial number as the position for the ROI. Please note that the position of the upper right corner for ROI detection is not fixed in acquired image coordinates due to the mechanical variations of an image acquisition system. Therefore, we labeled the correct upper right corner of the bounding box of the serial number manually in preparing a training data set. After training the ROI detection CNN, we generated the detected ROI regions. Using the detected ROI regions, we trained the classification CNN to classify characters in the extracted region simultaneously as done in a previous investigation [9]. We call this method multi-digit sequential (*md_seq*) method in this paper. To the best of our knowledge, this is the first attempt to decode all characters simultaneously for banknote serial number recognition.

The ROI detection CNN is trained by minimizing the following loss function for an input image:

(1) $$L_{det}={\left(\tilde{x}-x\right)}^{2}+{\left(\tilde{y}-y\right)}^{2},$$

where $\left(\tilde{x},\tilde{y}\right)$ are the labeled coordinates of the upper right corner of the input image and (x,y) are the predicted coordinates of the upper right corner. After training, using the predicted location from the ROI detection CNN, we extracted the bounding box image for the serial number from the input image and trained the classification CNN by minimizing the following loss function:

(2) $$L_{rec}=-\sum_{j}\sum_{k}p_{jk}\log q_{jk},$$

where $p_{jk}$ denotes the *true* probability of the *j*-th character in the input image and the *k*-th kind of character, and $q_{jk}$ is the corresponding predicted probability generated by the classification CNN. Note that $\sum_{k}p_{jk}=\sum_{k}q_{jk}=1$.

Although the *md_seq* method is straightforward to design, it may require long inference time as it has been shown that sequential operations require longer computation time than shallow networks [12,25]. Although the two sequential CNNs may work well to recognize the serial numbers of banknotes, we believe that it is possible to make the system faster by sharing features between the two CNNs. It was previously demonstrated that determining the possible region for an object and classifying the object by sharing the extracted features was successful for object detection [33]. It was also reported that shared feature extraction and simultaneous ROI detection and classification may greatly reduce computation time [34].

**Figure 6:**
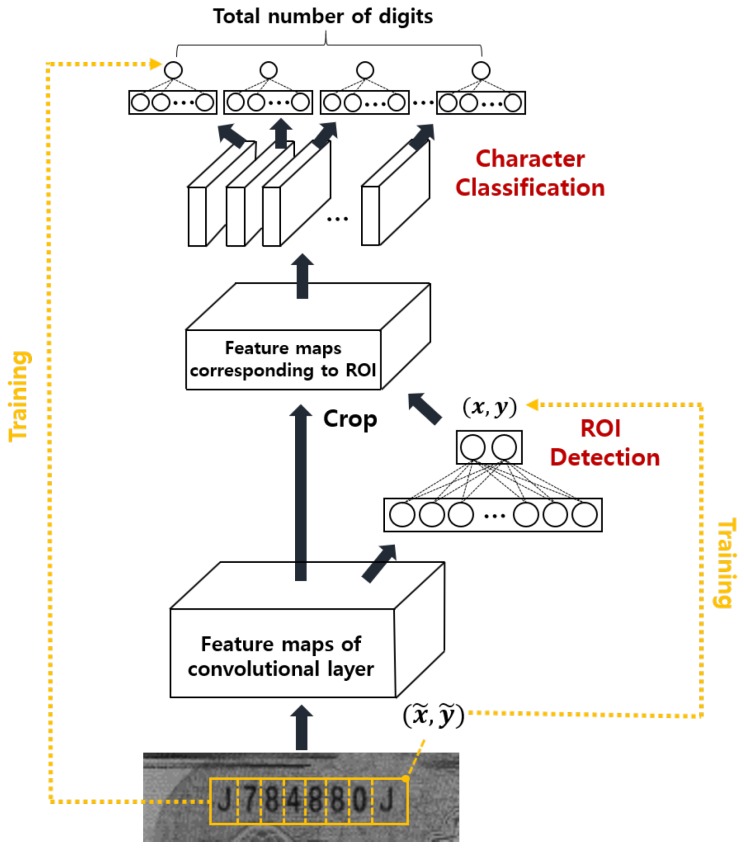
The joint ROI detection and classification system.

Based on these facts, we designed a deep-learning-based joint ROI detection and classification CNN as shown in Figure 6. In the figure, the ROI detection layer and character classification layer share convolutional layers that generate feature maps. Using the generated feature maps, the ROI detection layer predicts the upper right corner of the serial number region that is used to extract corresponding ROI regions of the feature maps. During training, the labeled location of the upper right corner was compared to the prediction (the right yellow line in Figure 6) and the training attempted to minimize the mean square error between the prediction and the labeled location. Using the predicted location, the ROI was extracted from the feature maps and the ROI became input for the character classification layer which classifies every digit simultaneously. Because certain regions in the feature map of the classification network correspond to one character, we only connected the fully connected layer for each character to the corresponding area in the feature map. This was possible because the size of the ROI and the number and kind of characters (i.e., numeric or alphabetic) are pre-determined by the kind of banknote. To train the classification network, we attempted to minimize the sum of the cross-entropy loss between the prediction and the labeled serial number at each digit (the left yellow line in Figure 6). Using this structure, we were able to detect all serial numbers simultaneously. We trained the entire deep-learning system shown in Figure 6 using labeled data for the ROI region in the input image and the *ground truth* serial number in the input image. Unlike existing methods, the method shown in Figure 6 recognizes all digits of the serial number simultaneously to achieve a fast computation time. We call this method multi-digit joint (*md_joint*) method. It has been reported that the inference time of one shallow network can be much faster than the sequential evaluation of one deep network even when there are a similar number of parameters due to parallel processing in the GPU [12].

The designed network can be trained using the labeled data by minimizing a loss function that consists of ROI detection error and classification error:

(3) $$L_{tot}=L_{rec}+\lambda L_{det},$$

where λ is a hyperparameter that determines weights for the two loss functions. Although the definition of $L_{rec}$ in (3) is the same as in (2), the input for computing the loss function is different. In (2), the loss is computed using the cropped image, while in (3) it is computed using the entire input image. Please note that the training of a joint ROI detection and character classification network can be more difficult than individual training of an ROI detection network and a character classification network because additional hyperparameters exist. Moreover, one may want to reduce the complexity of the system shown in Figure 6 by reducing the number of layers to achieve a faster computation time. We attempted to further reduce the computation time by applying knowledge distillation [12,24] which we discuss in the next section.

### 3.2. Knowledge Distillation

Model compression is a method for compressing a cumbersome deep-learning model into a small model [25]. Among several methods for model compression, knowledge distillation is a promising technique that transfers the trained knowledge of a cumbersome model to a simple model [12,24]. For classification and regression problems, a simple model can mimic the behavior of a cumbersome model by learning its logit values and predicted coordinates [12,24]. This approach has been shown to be more efficient than direct training of a simple model [12,24], partly because a small network may learn more information about the behavior of the cumbersome model.

Because fast real-time computation is important for banknote serial number recognition, we compressed the joint ROI detection and classification network using the knowledge distillation method by following the approach in [24]. We designed a network similar to that shown in Figure 6 but with a smaller number of fully connected layers. We call this method the multi-digit reduced joint (*md_rjoint*) method in this paper. We trained this simple model through knowledge distillation.

Suppose that the probability of a trained cumbersome model after a SoftMax layer is defined as follows [24]:

(4) $${\hat{c}}_{jk}=\frac{\exp(z_{jk})}{\sum_{k}\exp\left(z_{jk}\right)},$$

where $z_{jk}$ is the logit value of the cumbersome model for the *j*-th character in the input image and the *k*-th kind of character. Then we define the probability for knowledge distillation using the trained logit values as follows:

(5) $$c_{jk}=\frac{\exp(z_{jk}/T)}{\sum_{k}\exp\left(z_{jk}/T\right)},$$

where *T* is a constant parameter called temperature. We also define the probability of a small model for knowledge distillation with temperature as follows:

(6) $$s_{jk}=\frac{\exp(w_{jk}/T)}{\sum_{j}\exp\left(w_{jk}/T\right)},$$

where $w_{jk}$ is the logit value of the small model for the *j*-th character in the input image and the *k*-th kind of character. It has been shown that minimizing the cross-entropy between the cumbersome model and the small model is approximately equivalent to minimizing logit values between the two models when *T* is large [24]. Furthermore, it was demonstrated that proper selection of the *T* value is important because using larger values of *T* may degrade the performance of knowledge distillation when the logit value is small [24]. To accomplish knowledge distillation, we defined the loss function as follows:

(7) $$L_{kd}=-\sum_{j}\sum_{k}c_{jk}\log s_{jk}.$$

In addition to knowledge distillation, it has been shown that adding classification using a small network often improves performance [24]. To do that, we added another loss function, the classification cross-entropy, defined as follows:

(8) $$L_{cls}=-\sum_{j}\sum_{k}p_{jk}\log q_{jk},$$

where $p_{jk}$ is the labeled probability of the *j*-th character in the input image and the *k*-th kind of character, and $q_{jk}$ is the corresponding prediction from the small network with T=1 defined as follows:

(9) $$q_{jk}=\frac{\exp(w_{jk})}{\sum_{k}\exp\left(w_{jk}\right)}.$$

We also defined an ROI detection loss function to determine the serial number region as follows:

(10) $$L_{det}={\left(\hat{x}-x\right)}^{2}+{\left(\hat{y}-y\right)}^{2},$$

where $\left(\hat{x},\hat{y}\right)$ are the predicted coordinates from a cumbersome network and (x,y) are the predicted coordinates from our regression network. In addition to the loss functions described above, we used a well-known weight decay regularization function to prevent overfitting, defined as follows [35]:

(11) $$L_{wd}=w^{T}w,$$

where w is a column vector that contains every weight and bias in the system. Finally, we defined the total loss function as follows:

(12) $$L_{tot}=\lambda_{1}L_{det}+\left(1-\lambda_{1}\right)L_{kd}+\lambda_{2}L_{cls}+\lambda_{3}L_{wd},$$

where $\lambda_{1}$, $\lambda_{2}$, and $\lambda_{3}$ are weighting factors to determine weights for the four loss functions. We denote this method as the multi-digit reduced joint knowledge distillation (*md_rjoint_kd*) method. One must determine the hyperparameters $\lambda_{1}$, $\lambda_{2}$, $\lambda_{3}$, and *T* before training the small joint ROI detection and classification network.

### 3.3. Bayesian Optimization

Manual tuning of hyperparameters is cumbersome because there is a huge number of hyperparameter combinations. Moreover, since evaluating the performance of the joint ROI detection and classification system is time-consuming, a brute force search of the optimal parameters may require an extremely long computation time, possibly months. To solve this problem, we attempted to determine the optimal hyperparameters using the Bayesian optimization method [26] instead of manual tuning. Bayesian optimization-based methods have been shown to be effective for determining hyperparameters involved in machine learning [26,27,30,31,32].

Consider an optimization problem defined as follows:

(13) $$x^{*}=\underset{x\in X}{\mathrm{argmax}}f(x),$$

where X represents the parameter domain, x is the input parameter vector, and f(x) is an objective function that is not differentiable and difficult to compute. A typical example of such an objective function is the accuracy of a machine learning system that is neither differentiable with respect to searching hyperparameters nor easily evaluated. Please note that the evaluation of the loss function with a new hyperparameter usually requires significant computation because a deep-learning system needs to be trained with new hyperparameters. Therefore, conventional gradient-based optimization methods cannot be applied to determine hyperparameters.

The Bayesian optimization method can be effectively used when the objective function is non-differentiable, non-convex, and evaluations of the function are expensive [26,27,28]. It models the objective function f(x) as a random process and sequentially updates this model by observing data at $x_{n}$ (i.e., the output of the objective function with the input parameter vector $x_{n}$). After that, it selects new input parameters to be observed in the next evaluation by maximizing an acquisition function using the data observed so far [27,28]. The acquisition function is designed to be easier to evaluate and optimize than the original objective function. Typically, the acquisition function is designed by combining exploration and exploitation, which should be used appropriately to find the optimal parameters. Optimal parameters are in a region in which the uncertainty of the model is large (exploration) or where the prediction accuracy of the model is high (exploitation) [26].

To apply Bayesian optimization, we must choose a prior that can express assumptions about the objective function being optimized. We assumed that the objective function (which is the accuracy of the small machine learning system) is drawn from a Gaussian process as follows:

(14) $$f\left(x\right)∼GP(x;\mu \left(x\right),k\left(x,x^{\prime };\theta \right)),$$

where μ(x) and $k(x,x^{\prime };\theta )$ are a mean function and a covariance function, respectively. Since the results of machine learning systems are not deterministic, we also assumed that the observations are affected by Gaussian noise as follows:

(15) $$y=f\left(x\right)+\epsilon ,\;\epsilon ∼N\left(0,\sigma^{2}\right),$$

where **y** is observations and ϵ represents Gaussian noise with zero mean and $\sigma^{2}$ variance. In the Bayesian optimization method, covariance can be calculated using a kernel function that represents correlation according to the distance between two data points; the kernel function can be parameterized with smoothness parameters such as amplitude and length scales. We used automatic relevance determination (ARD) Matérn 5/2 kernel function, which is one of the most widely used kernels. The ARD Matérn 5/2 kernel showed faster convergence than other kernel functions in comparison in a previous investigation [27]. The ARD Matérn 5/2 kernel is defined as follows [26,27]:

(16) $$k\left(x,x^{\prime };\theta \right)=\theta_{0}^{2}exp\left(-\sqrt{5}r\right)\left(1+\sqrt{5}r+\frac{5}{3}r^{2}\right),$$

where $\theta_{0}$ is an amplitude and $r^{2}$ is defined as follows:

(17) $$r^{2}={\left(x-x^{\prime }\right)}^{T}\Lambda \left(x-x^{\prime }\right),$$

where Λ is a diagonal matrix of length scales $\theta_{i}$. We denote amplitude and length scales jointly by θ. These kernel parameters have a significant impact on determining the smoothness of the objective function. Therefore, it is important to determine the kernel parameters, which can be determined by maximizing a marginal likelihood function using the observed data as follows [26,30,36]:

(18) $$\hat{\theta }=\underset{\theta }{\mathrm{argmax}}\log p(y_{1:N};x_{1:N},\theta ),$$

where $\hat{\theta }$ is estimated kernel parameters, *N* is the number of data points observed to determine kernel parameters, and $\log p(y_{1:N};x_{1:N},\theta )$ is the marginal likelihood function which is defined as follows [26]:

(19) $$\log p(y_{1:N};x_{1:N},\theta )=-\frac{1}{2}{\left(y_{1:N}-\mu \left(x_{1:N}\right)\right)}^{T}{\left(K^{\theta }+\sigma^{2}I\right)}^{-1}\left(y_{1:N}-\mu \left(x_{1:N}\right)\right)-\frac{1}{2}\log|K^{\theta }+\sigma^{2}I|-\frac{n}{2}\log\left(2\pi \right),$$

where $K^{\theta }$ represents an N×N covariance matrix between observed input parameters as follows:

(20) $$K^{\theta }=\left[\begin{array}{ccc} k(x_{1},x_{1};\theta ) & \cdots & k(x_{1},x_{N};\theta )\\ \vdots & \ddots & \vdots \\ k(x_{N},x_{1};\theta ) & \cdots & k(x_{N},x_{N};\theta ) \end{array}\right].$$

The first term in (19) represents how well the model fits the observed data, while the second term represents the model complexity. As the correlation of the data increases, the model becomes smoother and the determinant becomes smaller. We determined the kernel parameters that best represented the observed data by maximizing the marginal likelihood function; when maximizing the marginal likelihood function, we applied an iterative optimization method since the gradient of function has no closed-form solution.

Since we assumed that the objective function is drawn from a Gaussian process, the output at any one point x follows a Gaussian distribution with mean and variance. However, the mean and variance are not known to us; the Bayesian optimization method estimates the mean and variance from prior mean and covariance via Bayesian posterior updating using the observed data $x_{1:n}$ and $y_{1:n}$. The posterior mean is defined as follows [26]:

(21) $$\mu_{n}\left(x;x_{1:n},y_{1:n}\right)=\mu \left(x\right)+k^{\hat{\theta }}{\left(x\right)}^{T}{\left(K^{\hat{\theta }}+\sigma^{2}I\right)}^{-1}\left(y_{1:n}-\mu \left(x_{1:n}\right)\right),$$

where x is an arbitrary input parameter vector contained in the parameter domain X, *n* is the number of observed data points that should be greater than *N*, $\mu_{n}\left(x;x_{1:n},y_{1:n}\right)$ is the posterior mean that represents the prediction of the model at the point x, $K^{\hat{\theta }}$ is the n×n covariance matrix, and $k^{\hat{\theta }}\left(x\right)$ represents an n×1 covariance vector between an arbitrary input parameter vector x and observed input parameters $x_{1:n}$ as follows:

(22) $$k^{\hat{\theta }}\left(x\right)={\left[\begin{array}{cccc} k(x,x_{1};\hat{\theta }) & k(x,x_{2};\hat{\theta }) & \cdots & k(x,x_{n};\hat{\theta }) \end{array}\right]}^{T}.$$

The posterior variance is also defined as follows [26]:

(23) $$\sigma_{n}^{2}\left(x;x_{1:n}\right)=k^{\hat{\theta }}\left(x,x\right)-k^{\hat{\theta }}{\left(x\right)}^{T}{\left(K^{\hat{\theta }}+\sigma^{2}I\right)}^{-1}k^{\hat{\theta }}\left(x\right),$$

where $\sigma_{n}^{2}\left(x;x_{1:n}\right)$ is the posterior variance that represents uncertainty of the model at the point x. We simply denote the posterior mean and variance by $\mu_{n}\left(x\right)$ and $\sigma_{n}^{2}\left(x\right)$, respectively.

To update the model sequentially, we determined the next input parameter vector to be observed by maximizing the acquisition function as follows [26]:

(24) $$x_{n+1}=\underset{x}{\mathrm{argmax}}\alpha_{n}\left(x;\mu_{n}\left(x\right),\sigma_{n}\left(x\right)\right),$$

where $\alpha_{n}\left(x;\mu_{n}\left(x\right),\sigma_{n}\left(x\right)\right)$ is the best-known expected improvement (EI) acquisition function that incorporates the amount of improvement which is defined as follows [26]:

(25) $$\begin{array}{cc} \alpha_{n}\left(x;\mu_{n}\left(x\right),\sigma_{n}\left(x\right)\right) & =E[I(x)]\\ & =E[\max\left(f_{n}\left(x\right)-\tau_{n},0\right)]\\ & =\left(\mu_{n}\left(x\right)-\tau_{n}\right)\Phi \left(\frac{\mu_{n}\left(x\right)-\tau_{n}}{\sigma_{n}\left(x\right)}\right)+\sigma_{n}\left(x\right)\phi \left(\frac{\mu_{n}\left(x\right)-\tau_{n}}{\sigma_{n}\left(x\right)}\right), \end{array}$$

where I(x) is the improvement at **x**, Φ is the standard normal cumulative distribution function, ϕ is the standard normal probability density function, and $\tau_{n}$ denotes the maximum value of observations $y_{1:n}$ (i.e., accuracy). Although there are various acquisition functions, many studies related to Bayesian optimization focus on the EI function, since it does not require any tuning parameters and can balance exploitation (the first term in (25)) and exploration (the second term in (25)) properly [26,27,30].

**Figure 7:**
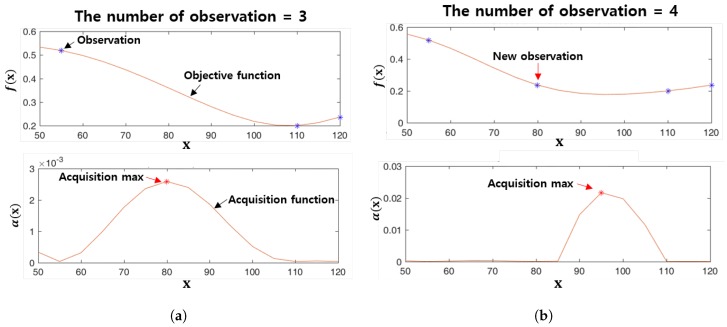
The Bayesian optimization procedure. (**a**) The number of observations is 3 (**b**) The number of observations is 4.

Figure 7 shows the Bayesian optimization procedure. Figure 7a shows the objective function estimated by observing three data points (the blue points) and the acquisition function calculated using the posterior mean and variance from the estimated objective function. In Figure 7a, the red point indicates the point at which the acquisition function is maximized, and the input parameter x at this point is selected as the next input parameter to be observed as shown in the top of Figure 7b. Figure 7b repeats the process shown in Figure 7a using four observations.

## 4. Experimental Results

We evaluated the performance of the four multi-digit serial number recognition methods (*md_seq*, *md_joint*, *md_rjoint*, and *md_rjoint_kd* methods) in comparison with the conventional single digit recognition methods (*sd_cnn* method and *sd_cnn_svm* method). For the *sd_cnn* and *sd_cnn_svm* methods, we used ROIs detected by the *md_seq* method. We determined hyperparameters for the *md_rjoint_kd* method using Bayesian optimization. Although there are four different hyperparameters for knowledge distillation *T*, $\lambda_{1}$, $\lambda_{2}$, and $\lambda_{3}$ in (5) and (12), we fixed $\lambda_{3}$ to 0.02 to reduce the dimensionality of the parameters and determined the remaining three parameters using Bayesian optimization. The *md_joint* and *md_rjoint* methods also need to determine the hyperparameter λ which controls weighting between two loss functions in (3). We determined λ to be 0.01 through manual tuning.

Table 1 shows the network structure of each method. We implemented all CNN models using the TensorFlow library (Google, Inc.) and the SVM models using the Thunder SVM library [37] which is a fast SVM library that uses GPU. We trained all SVM models with the radial basis function (RBF) kernel and one-vs-one [38] in classification. All CNN models were trained by Adam optimizer with an initial learning rate of 1 × 10${}^{-4}$, a batch size of 64, and an epoch of 130. We set the momentum parameters $\beta_{1}$ and $\beta_{2}$ for Adam optimizer to 0.9 and 0.999, respectively. We also randomly selected 5% of the training data as validation data in every epoch to monitor the performance of the model and whether overfitting occurred during training. We confirmed that the accuracy of both the training set and the validation set converged during training. However, we did not use the validation data for early stopping.

**Figure 8:**
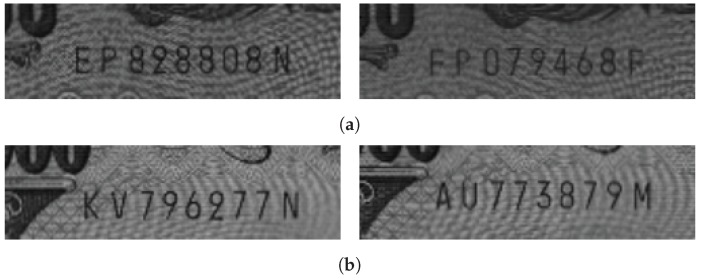
Typical images from set A. (**a**) 1000 Yen (**b**) 5000 Yen.

**Figure 9:**
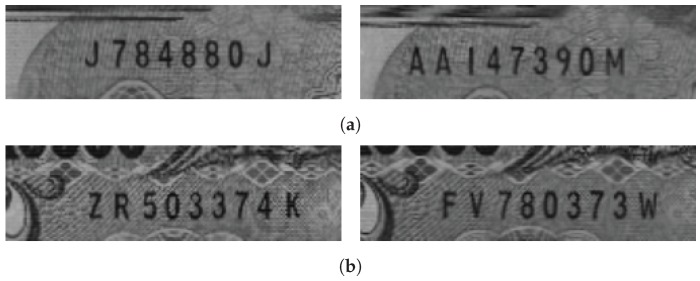
Typical images from set B. (**a**) 2000 Yen (**b**) 10,000 Yen.

**Figure 10:**
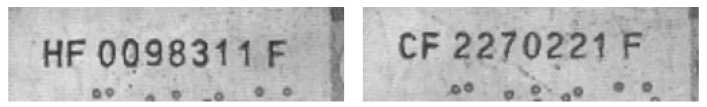
Typical images from set C: 10,000 Won.

**Figure 11:**
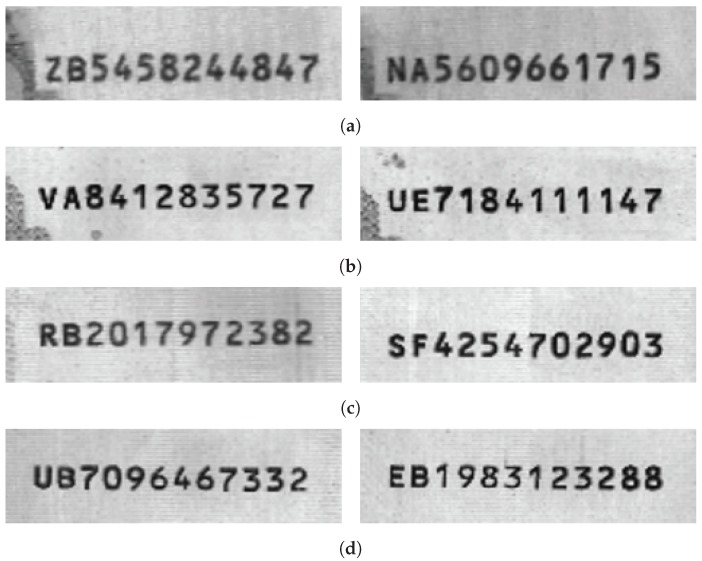
Typical images from set D. (**a**) 5 Euro (**b**) 10 Euro (**c**) 20 Euro (**d**) 50 Euro.

We prepared four data sets including Japanese Yen, Korean Won, and Euro banknotes to evaluate the performance of each method. The first data set (A) contained banknotes of 1000 Yen and 5000 Yen and the second data set (B) contained banknotes of 2000 Yen and 10,000 Yen. We divided the images of the Japanese banknotes into two data sets based on the similarity of the images. The third data set (C) contained banknotes of 10,000 Won and the fourth data set (D) contained banknotes of 5 Euro, 10 Euro, 20 Euro, and 50 Euro. Based on the number of ROI images, set A had 7680 training data and 1009 test data; set B had 9566 training data and 1880 test data. Set C had 9024 training data and 1910 test data; set D had 7232 training data and 1052 test data. Figure 8, Figure 9, Figure 10 and Figure 11 show images from the four sets, respectively. As shown in the figures, the fonts of the characters and the backgrounds of the four sets are different. We trained the deep-learning systems for each data set separately to yield accurate recognition of each banknote. All experimental data sets are available at https://github.com/ejeong93/SNRdataset.

Table 2 shows the recognition results from the six methods using the four data sets. We trained each model 5 times and reported test accuracies of the test result averages. However, we trained the *md_seq* method only once because one model was required for knowledge distillation. To calculate the test accuracy, we counted the number of test images in which all digits were correctly classified and then divided it by the total number of test images.

In Table 3, we also reported the inference time of each method. This was computed as the average of 100 times for 64 arbitrary images (the batch size) from each data set using a PC with Intel(R) Core(TM) i7-7700 CPU and NVIDIA GeForce GTX 1060. As shown in the tables, the *sd_cnn*, *sd_cnn_svm*, and *md_seq* methods showed similar accuracies while the inference time of the *md_seq* method was about 12.10 milliseconds (32.99%) shorter than the *sd_cnn* method and about 24.72 milliseconds (50.14%) shorter than the *sd_cnn_svm* method. The *sd_cnn_svm* method was the slowest because it includes the feature extraction process from the trained CNN model. The *md_joint* method was faster than the *md_seq* method while the performance was slightly worse than the *md_seq* method. We suspect that this is because it is more difficult to train a joint system. Although the *md_rjoint* method was faster than the *md_joint* method, the accuracy of the *md_rjoint* method was worse than other methods. The *md_rjoint_kd* method was the fastest (equal to *md_rjoint*) method with high accuracy. The inference time of the *md_rjoint_kd* method was about 16.22 milliseconds (44.22%) shorter than the *sd_cnn* method and about 28.84 milliseconds (58.50%) shorter than the *sd_cnn_svm* method and about 4.12 milliseconds (16.76%) shorter than the *md_seq* method. Although the *md_rjoint_kd* method had the same network structure as the *md_rjoint* method, the *md_rjoint_kd* method showed surprisingly higher accuracy because it was effectively trained to mimic the behavior of the *md_seq* method which had high accuracy. In addition, the hyperparameters for knowledge distillation were appropriately determined to acquire high accuracy using Bayesian optimization. Please note that 58.50%, 44.22% and 16.76% of speed improvements can be very important in real-time applications that use an embedded system. In addition, the *md_rjoint_kd* method using knowledge distillation techniques for set B and D performs even better than the *md_seq* teacher model, as shown in Table 2. Please note that this is not surprising since it was reported previously that a shallow model may perform better than a teacher model [12].

Table 4 shows the hyperparameters determined using the Bayesian optimization method. As discussed above, the Bayesian optimization method requires choosing three hyperparameters: temperature *T*, from 1 to 1000; weighting factor $\lambda_{1}$, from 0.1 to 0.9; and weighting factor $\lambda_{2}$, from 0.01 to 0.9. We also defined the objective function as the mean accuracy averaged five times and evaluated the *md_rjoint_kd* method 50 times to determine the hyperparameters for knowledge distillation. Among the 50 evaluations, eight observations were selected to determine initial kernel parameters by minimizing the negative marginal likelihood function using the “minimize” function of the SciPy library. To apply Bayesian optimization, we also used the initial mean value of 0.5 (since accuracy is in the range of 0 to 1), an observation noise variance of $10^{-6}$, the ARD Matérn 5/2 kernel function, and the EI function for the acquisition function. We terminated the search for hyperparameters with Bayesian optimization if the already observed parameters were selected as the parameters to be observed in the next evaluation.

**Figure 12:**
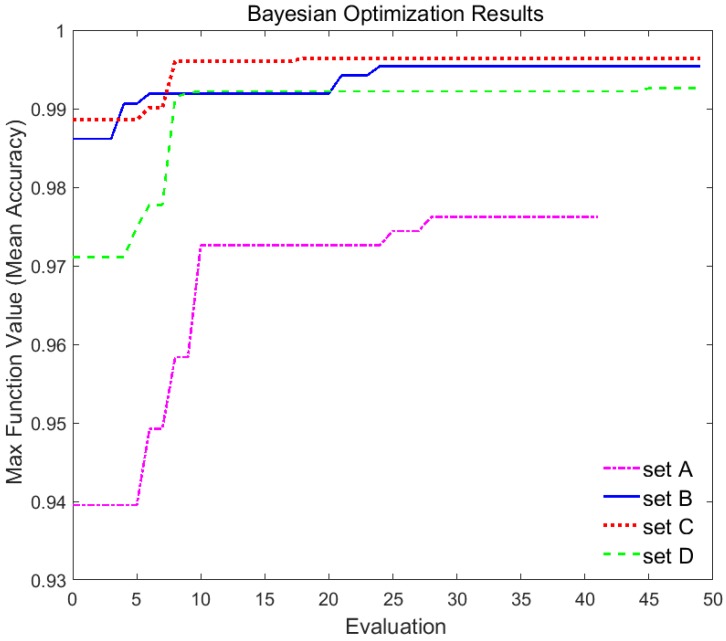
Bayesian optimization results for data set.

Figure 12 shows a graph of the maximum mean accuracy found with the Bayesian optimization method over the number of evaluations for each data set. Please note that Bayesian optimization is the method to find hyperparameters when the objective function is maximized. As shown in Figure 12 and Table 2, the Bayesian optimization method found the hyperparameters with an accuracy of 97.62% using only 28 observations for set A and with an accuracy of 99.54% using only 24 observations for set B. Figure 12 also shows this method found the hyperparameters with an accuracy of 99.64% using only 18 observations for set C and with an accuracy of 99.26% using 45 observations for set D. Moreover, the accuracy of the *md_rjoint_kd* method for set B and D was superior to the teacher model, the *md_seq* method.

## 5. Discussion

We investigated knowledge distillation and a Bayesian optimization method for improving the performance of machine learning-based serial number recognition for Japanese Yen, Korean Won, and Euro banknotes. If one wants to apply the proposed method for other banknotes, it is necessary to modify the size of the ROI and the number and kind of characters which are pre-determined by the kind of banknotes. In addition, re-training the machine learning model using new banknotes is necessary.

In our experiments, the joint recognition method was faster than the sequential method while showing similar accuracy, which implies that it is possible to share convolution layers for ROI detection and serial number recognition. Although the reduced joint model trained by traditional supervised learning showed very poor performance, it was possible to train the reduced model to have performance comparable to the sequential method using knowledge distillation. We believe that this was possible because the reduced system learns rich behaviors of the sequential model [12,24].

In this paper, we assumed that banknote classification is perfectly performed before serial number recognition. If banknote classification is wrong, serial number recognition performance may be degraded because our recognition methods in this paper use results of the banknote classification. One may conceive of a joint method for banknote classification and serial number recognition which is a subject for future study.

Since banknote recognition is based on images acquired using image sensors, sensor signal pre-processing methods may affect the performance of the recognition. Investigation of pros and cons of different sensor signal processing methods for banknote recognition is also a subject for future study.

In addition, one may argue that there exist better serial number recognition methods than the proposed method. We do not intend to argue that the proposed method is superior to every state-of-the-art method. The focus of this research is the investigation of the usefulness of knowledge distillation and the Bayesian optimization method for a CNN-based joint ROI detection and multi-digit serial number recognition system. Comparison of the proposed method with other state-of-the-art methods will be considered for future work.

## 6. Conclusions

We propose a fast machine learning-based serial number recognition system for banknotes. For fast computation, the proposed method simultaneously determines the region of interest for the serial number and classifies the characters in the serial number. Moreover, we apply knowledge distillation to reduce the complexity of the proposed method. We determine several hyperparameters involved in knowledge distillation using the Bayesian optimization method. We verify the usefulness of the proposed method in experiments using Japanese Yen, Korean Won, and Euro banknotes.

## Figures and Tables

**Table 1:** The network structure for each method.

	ROI Detection	Character Classification
***sd_cnn***	conv1-pool1-conv2-conv3-fc1-fc2	conv4-pool2-conv5-conv6-fc3-fc4
***sd_cnn_svm***	conv1-pool1-conv2-conv3-fc1-fc2	conv4-pool2-conv5-conv6-fc3-SVM
***md_seq***	conv1-pool1-conv2-conv3-fc1-fc2	conv4-pool2-conv5-conv6-fc3-fc4
***md_joint***	conv1-pool1-conv2-conv3-fc1-fc2	conv1-pool1-conv2-conv3-fc3-fc4
***md_rjoint***	conv1-pool1-conv2-conv3-fc1	conv1-pool1-conv2-conv3-fc2
***md_rjoint_kd***	conv1-pool1-conv2-conv3-fc1	conv1-pool1-conv2-conv3-fc2

Conv, pool, and fc stand for convolutional layer, max pooling layer, and fully connected layer, respectively.

**Table 2:** Performance of all methods.

		*sd_cnn*	*sd_cnn_svm*	*md_seq*	*md_joint*	*md_rjoint*	*md_rjoint_kd*
**Accuracy (%)**	**set A**	97.29	96.33	98.02	97.03	89.81	97.62
	**set B**	98.23	97.12	99.26	98.69	97.70	99.54
	**set C**	99.53	99.13	99.69	98.12	85.59	99.64
	**set D**	99.26	99.17	99.24	98.86	88.35	99.26

**Table 3:** Inference time (msec).

	*sd_cnn*	*sd_cnn_svm*	*md_seq*	*md_joint*	*md_rjoint*	*md_rjoint_kd*
ROI detection	18.31	18.43	17.78	22.02	20.50	20.46
Character classification	18.37	30.87	6.80			
Total	36.68	49.30	24.58	22.02	20.50	20.46

**Table 4:** Hyperparameters determined from Bayesian optimization.

	Set A			Set B			Set C			Set D		
	T	$\lambda_{1}$	$\lambda_{2}$	T	$\lambda_{1}$	$\lambda_{2}$	T	$\lambda_{1}$	$\lambda_{2}$	T	$\lambda_{1}$	$\lambda_{2}$
***md_rjoint_kd***	729	0.4	0.88	33	0.1	0.88	949	0.1	0.01	981	0.1	0.88
